# Immunohistochemical detection of MUC5AC and MUC5B mucins in ferrets

**DOI:** 10.1186/s13104-023-06388-x

**Published:** 2023-06-22

**Authors:** David K. Meyerholz, Mariah R. Leidinger, J. Adam Goeken, Thomas R. Businga, Sebastian Vizuett, Allison Akers, Idil Evans, Yan Zhang, John F. Engelhardt

**Affiliations:** 1 Department of Pathology, Carver College of Medicine, University of Iowa, Iowa City, IA 52242 USA; 2 Department of Anatomy and Cell Biology, Carver College of Medicine, University of Iowa, Iowa City, IA 52242 USA

**Keywords:** Cystic fibrosis, Ferret, Immunohistochemistry, Lung, Mucin, Mucus, MUC5AC, MUC5B, Tissue, Pathology

## Abstract

**Objective:**

Cystic fibrosis (CF) is a genetic condition that causes abnormal mucus secretions in affected organs. MUC5AC and MUC5B are gel-forming mucins and frequent targets for investigations in CF tissues. Our objective was to qualify MUC5AC and MUC5B immunohistochemical techniques to provide a useful tool to identify, localize and interpret mucin expression in ferret tissues.

**Results:**

MUC5AC and MUC5B mucins were detected most commonly in large airways and least in small airways, consistent with reported goblet cell density in airway surface epithelia. We evaluated whether staining method affected the detection of goblet cell mucins in serial sections of bronchial surface epithelia. Significant differences between stains were not observed suggesting common co-expression MUC5AC and MUC5B proteins in goblet cells of airway surface epithelia. Gallbladder and stomach tissues are reported to have differential mucin enrichment, so we tested these tissues in wildtype ferrets. Stomach tissues were enriched in MUC5AC and gallbladder tissues enriched in MUC5B, mucin enrichment similar to human tissues. Mucin immunostaining techniques were further qualified for specificity using lung tissue from recently generated *MUC5AC*^*−/−*^ and *MUC5B*^*−/−*^ ferrets. Qualified techniques for MUC5AC and MUC5B immunohistochemistry will be useful tools for mucin tissue studies in CF and other ferret models.

## Introduction

Cystic fibrosis (CF) is a life-limiting condition caused by mutations in the CF transmembrane conductance regulator (CFTR) [1, 2]. Clinical disease can begin before birth and produces lesions in several organ systems including: respiratory tract, gastrointestinal tract, skin, and reproductive tract [3–5]. Mouse models were developed by 1992, but these lacked significant phenotypes in key organs, thus accelerating the search for other novel animal models. With the advent of somatic cell nuclear transfer technology, the CF pig [6]and CF ferret [7] models were some of the first new animal models developed. Phenotypic analysis of the CF ferret model was reported in 2010 and has since been useful for study of lung, gastrointestinal, and pancreatic disease as well as novel treatment strategies [7–11].

Mucins are high molecular weight glycoproteins that provide the characteristic viscoelastic features of mucus. In the respiratory tract, MUC5AC (goblet cells) and MUC5B (goblet in surface epithelia and mucous cells of submucosal glands) are the major gel-forming mucins [12]. As part of mucociliary clearance, thin strands of secreted mucus sweep airways of inhaled debris and pathogens [13]. CF mucus is abnormal and described as thick, sticky and tenacious, features that contribute to its pathological role in disease development [12, 14]. Qualified immunohistochemical staining for MUC5AC and MUC5B can augment tissue studies for CF mucus [15].

The aims of the current study were to qualify the MUC5AC and MUC5B immunostaining techniques through use of control ferret tissues including the lung.

## Main text

### Methods

Archival paraffin-embedded tissue blocks from wildtype (WT) ferrets or those with ongoing research projects at the University of Iowa. All studies were performed under the approval and guidance of the University of Iowa Animal Care and Use Committee and followed all pertinent federal/national/international standards of care. Adult WT ferrets (1–2 years of age, n = 2–3 per sex) were used to evaluate mucin expression in select tissues (lung, sinonasal cavity, pancreas, gallbladder, stomach) that are relevant to CF research. Additionally, lung tissue of recently developed *MUC5AC*^*−/−*^ (male, n = 1, > 1 yr age) and *MUC5B*^*−/−*^ (female, n = 1, > 1 yr age) ferrets were obtained from an ongoing separate phenotypic study of these novel ferret models. Disruption of the two mucin genes was achieved using Cas9/gRNA ribonuclear protein complexes into ferret zygotes followed by adaptive transfer into pseudo-pregnant jills [16]. These exclusive, few lung tissues from novel mucin models were used to help qualify the specificity of mucin immunohistochemistry in the current study and any additional data about the generation or characterization of these models will be published in a separate study.

In tissue sections (~ 4 µm), diastase-pretreated periodic acid Schiff (dPAS) histochemical stain was applied to detect and localize mucus in tissues [17]. Baseline protocol parameters for evaluation of immunohistochemical staining for MUC5AC and MUC5B were guided from previous reports in CF models [15, 18–20]. The primary antibody concentration was preliminarily tested via a panel of concentrations (1:250, 1:500, 1:1000, 1:2000) to evaluate for staining of known positive cells (e.g. goblet cells) and absence of staining in off target cells to yield the initial baseline techniques used in this study. For both mucins, tissues were exposed to heat-induced epitope retrieval (citrate buffer pH 6.0, 110 °C × 15 min), followed by primary antibody for MUC5AC (mouse monoclonal 1:500 × 1 h, clone 45M1, #ab3649, Abcam, Waltham, MA, USA) or MUC5B (rabbit polyclonal 1:1000 × 20 min, #HPA008246, Sigma Aldrich, St. Louis, Mo, USA). Secondary kits of Mouse EnVision + and Rabbit Envison (Dako North America, Inc., Carpentaria, CA, USA) were respectively applied followed by 3,3′-Diaminobenzidine as chromogen and Harris hematoxylin as counterstain. Qualification of the immunostaining was primarily evaluated in gallbladder, stomach and lung tissues of wildtype ferrets (see results section). Anatomic definitions for airway size in ferret lungs were as follows: large bronchi (50–100% circumferential cartilage), small bronchi (< 1–50% circumferential cartilage), bronchioles (no cartilage in airway wall).

Representative high-resolution digital images were collected and analyzed (BX53 microscope, DP73 digital camera and CellSens Dimension Software, Olympus). Area of immunostaining relative to total area of airway surface epithelium produced the area fraction of immunostaining. These results were statistically analyzed with either two-way ANOVA or Kruskal–Wallis test as warranted using Prism software (Graphpad, Sand Diego, CA, USA). Airway epithelia height (as a metric of airway caliber) [21] and mucin expression were analyzed using Spearman correlation to define r and P values (significance defined as P < 0.05).

## Results

**Fig. 1:**
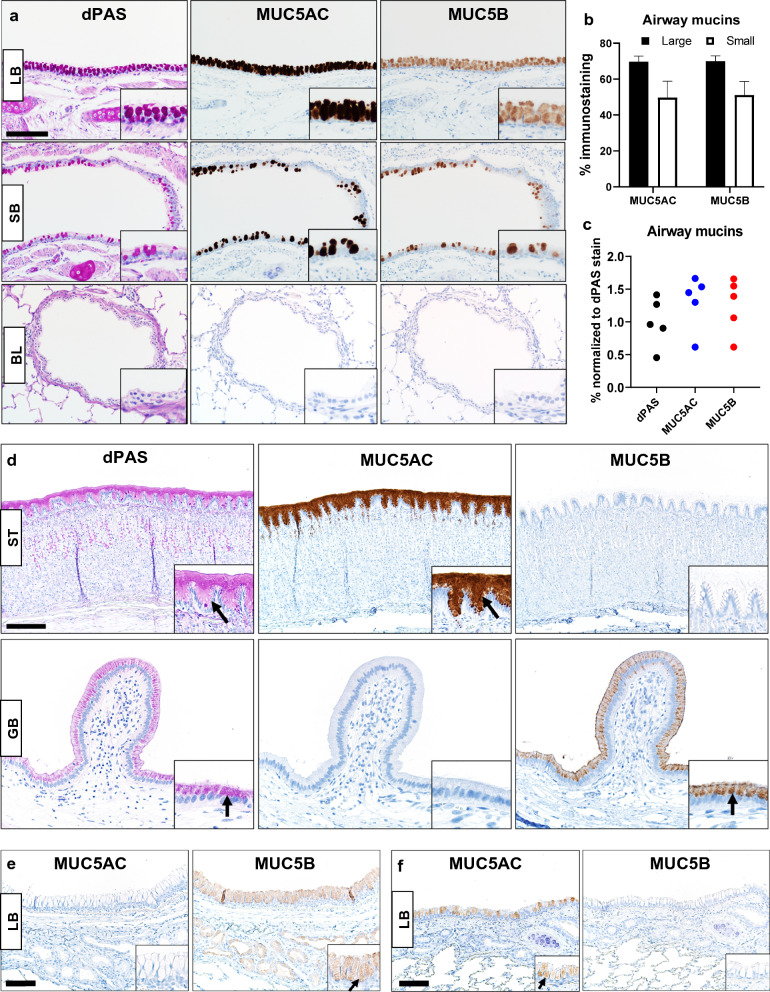
Mucin detection in ferret tissues. **a** Representative mucin detection (insets) by dPAS, MUC5AC and MUC5B in surface epithelia of large bronchi (LB) or small bronchi (SB) and bronchioles (BL) from WT ferrets (bar = 130 µm). **b** Evaluation of MUC5AC and MUC5B immunostaining in surface epithelia of large and small bronchi (N = 5 WT ferrets) using digital image analysis. Airway size was a significant factor for the variance in mucin detection (P = 0.0072, Two-way ANOVA). **c** Comparison of dPAS, MUC5AC and MUC5B in serial sections of WT bronchus (N = 5 WT ferrets) showed no significant differences in mucin detection between stain method (P = 0.2938) Kruskal–Wallis test). **d** Representative images of differential mucin expression (arrows and insets) in WT ferret stomach (ST, bar = 170 µm) and gallbladder (GB, bar = 85 µm). dPAS, MUC5AC and MUC5B. **e** Mucin detection (arrow and insets) in a large bronchus (LB) from a *MUC5AC*^*−/−*^ ferret (N = 1). Surface epithelia and submucosal glands were MUC5B + confirming the tissue was viable for immunostaining. MUC5AC immunostaining was absent from the surface epithelia, and this was consistent with the ferret’s genotype, bar = 85 µm. **f** Mucin detection (inset and arrows) in a large bronchus (LB) from a *MUC5B*^*−/−*^ ferret (N = 1). The surface epithelia of the bronchus was MUC5AC+ confirming the tissue was viable for immunostaining, but it was MUC5B- consistent with the tissue genotype, bar = 85 µm

Positive and negative cellular or tissue expression of protein targets are useful to qualify the specificity and utility to immunohistochemical techniques [22, 23]. We evaluated mucin detection in bronchi (large and small) and bronchioles of WT ferret lungs. Mucins were detected more abundantly in large bronchi than small bronchi, but detection in bronchioles was rare to absent (Fig. 1a). Digital image analysis of airway mucins showed that the size of ferret bronchi significantly influenced mucin detection (P = 0.0072, two-way ANOVA, Fig. 1b), indicating that MUC5AC and MUC5B were more prevalent in larger than smaller bronchi. Correlation analysis of the airway mucin expression and airway epithelia height (a surrogate marker of airway caliber) demonstrated a significant relationship for MUC5AC (r = 0.6606, P = 0.044, Spearman correlation) and MUC5B (r = 0.6848, P = 0.035, Spearman correlation). In serial sections of bronchi surface epithelium, dPAS, MUC5AC and MUC5B techniques were digitally analyzed. We saw no significant differences between these mucin stains (Fig. 1c, P = 0.2938, Kruskal–Wallis test), suggesting MUC5AC and MUC5B have common co-expression in goblet cells.

Healthy gallbladder and stomach tissues have been reported to have distinct tissue enrichment of MUC5AC in stomach and MUC5B in gallbladder [24, 25]. We evaluated stomach and gallbladder tissues from WT ferrets to see if similar mucin-specific enrichment was observed. MUC5AC immunostaining was detected in ferret stomach but was absent in ferret gallbladder. In contrast, MUC5B immunostaining was detected in ferret gallbladder, but absent in ferret stomach (Fig. 1d). These data parallel mucin enrichment observed in humans and several animal models (Table 1) [24–36].

**Table 1:** Species comparison of mucin enrichment in healthy gallbladder and stomach tissues

Species	Gallbladder	Stomach
Human	MUC5B high [24, 27] MUC5AC low [26, 28]	MUC5AC high [25] MUC5B low (absent except in fetal development or disease) [26]
Ferret	MUC5B high, MUC5AC low (see results section)	MUC5AC high, MUC5B low (see results section)
Pigs	MUC5B high [36] MUC5AC low [36]	MUC5AC high [31, 33]
Sheep	NA	MUC5AC high [30]
Rabbits	NA	MUC5AC high [31]
Rat	Rats do not have gallbladders	MUC5AC high [31, 32]
Mice	MUC5B high [34]	MUC5AC high [29, 35]

"High" = Moderate to strong expression, "Low" = Minor to lack of expression

Immunohistochemical techniques can also be qualified by testing tissues that lack antigen/epitope expression due to genomic editing. We acquired access to rare bronchial tissues from two novel models (a *MUC5AC*^*−/−*^ ferret and a *MUC5B*^*−/−*^ ferret) that are being phenotypically characterized for an ongoing separate study. We evaluated serial sections of a bronchus from a *MUC5AC*^−/−^ ferret and both the surface epithelium and submucosal glands had MUC5B + immunostaining, but MUC5AC immunostaining was negative consistent with the tissue genotype (Fig. 1e, see Fig. 1a for reference of WT staining). We then evaluated a bronchus from a *MUC5B*^−/−^ ferret. The surface epithelium was positive for MUC5AC immunostaining but it was negative for MUC5B consistent with expected expression patterns the model (Fig. 1f, see Fig. 1a for reference of WT staining).

**Fig. 2:**
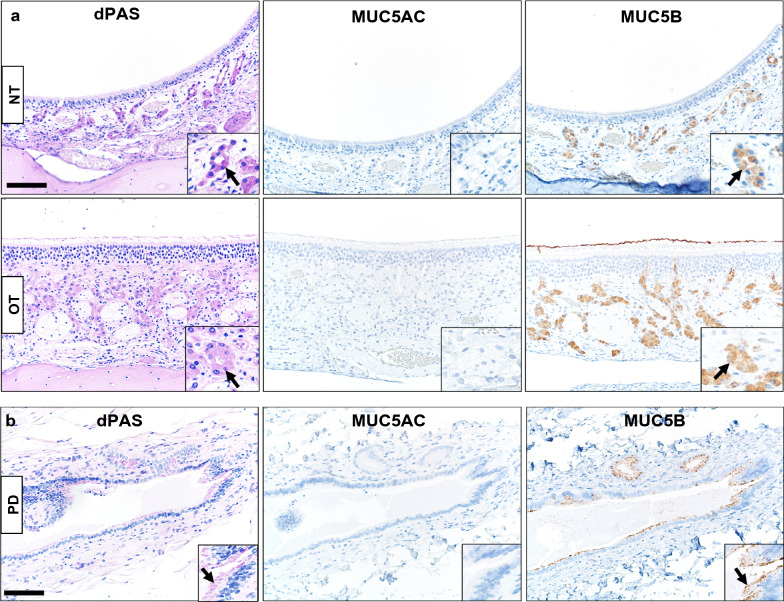
Representative mucin detection in WT tissues. **a** dPAS + and MUC5B + mucin detection (arrows and insets) in glands of nasal (NT) and olfactory (OT) tissues from WT ferret sinonasal cavity, but the glands were MUC5AC−, bar = 85 µm. **b** dPAS + and MUC5B + (arrows and insets) mucin detection in a large pancreas duct (PD) from a WT ferret, but duct epithelia were MUC5AC−, bar = 85 µm

We then applied the MUC5AC and MUC5B immunostaining on two other WT ferret tissues that might be of interest for study in the CF, specifically sinonasal cavity and pancreas. In the sinonasal cavity, the glands of the respiratory epithelia and Bowman glands of olfactory epithelia were dPAS + and MUC5B + while MUC5AC immunostaining was lacking (Fig. 2a). These findings parallel a report of healthy human nasal glands and Bowman glands with MUC5B + expression and minimal/lack of MUC5AC immunostaining [37, 38]. In the pancreas, large secretory ducts had evidence of dPAS + mucins in mucous cells and these sites paralleled with MUC5B + immunostaining but was negative for MUC5AC (Fig. 2b). Healthy human pancreas ducts are reported to have MUC5B expression and lack MUC5AC expression, but both may be present in diseased pancreas (e.g. cancer) [39].

In all of the studies, we did not see evidence for overt sex-specific differences in localization or distribution of mucin expression.

## Discussion

Immunohistochemical techniques can be qualified by multiple approaches through use of appropriate controls [23, 40, 41]. In this current study, our control cell/tissue were defined from previous reports of airway mucin expression patterns in lungs, known anatomic / tissue enrichment differences, and use of gene-edited tissues. We were able apply these mucin immunohistochemical techniques in ferret tissues to define mucin expression for CF-relevant tissues such as lung, gallbladder, stomach, pancreas and sinonasal cavity and show its assessment through digital image analysis [9, 20, 42]. Mucin expression patterns in healthy tissues are useful controls to clarify expression changes that can appear in diseased tissues, such as prospective studies on ferret tissues to evaluate CF mucins [7, 28, 39, 43, 44]. Even so, mucins may be a pertinent parameter for study in ferret tissues modelling other diseases such as transplantation rejection [45], chronic obstructive pulmonary disease [46], COVID19 [21, 47], influenza [48], filoviruses [49] and cancer [50] to name a few.

For quality control, immunohistochemical techniques should be initially qualified before use in investigative studies [22, 51]. Multiple layers of immunohistochemical qualification (as seen in this study) provide added confidence in the use of these mucin detection techniques for tissue studies of CF and other ferret models. Additionally, requalification of any immunohistochemistry protocol is also recommended after changes in pre-analytic tissue factors or protocol reagents (lots, reagents, etc.) as these can affect the qualities of the final immunostaining [52, 53]. Our qualification of MUC5AC and MUC5B techniques in ferret tissues, use of control tissues and application of digital image analysis confirm that these mucin detection techniques will be vital tools to identify, localize and interpret mucin expression. Additionally, these tools will be useful in future studies to analyze the spatial and cellular expression of mucins in ferret lungs and compare to human lungs [54].

## Limitations

This study is not without potential limitations. First, we studied select archival tissues from adult ferrets, but we cannot fully assume that our results will be directly applicable to other ferret tissues/organs or other ferret breeds/strains. Second, we focused our evaluation of healthy tissues, so we cannot rule out that diseased tissues with inflammation or remodeling changes might display differences in cellular localization or intensity of mucins. Lastly, it is well-recognized that pre-analytic factors (tissue handling, fixation quality, etc.) can greatly influence immunostaining and digital image analysis [22, 41, 55]. Thus, differences in pre-analytical could feasibly produce minor lab-to-lab variations in immunostaining results.

## Data Availability

The datasets used and/or analyzed during the current study are available from the corresponding author on reasonable request.

## Abbreviations

BL: Bronchiole
CF: Cystic fibrosis
CFTR: Cystic fibrosis transmembrane conductance regulator
COVID19: Coronavirus disease of 2019
GB: Gallbladder
LB: Large bronchi
NT: Nasal tissue
OT: Olfactory tissue
SB: Small bronchi
ST: Stomach
dPAS: Diastase-pretreated periodic acid Schiff
WT: Wildtype
